# Sexual Compatibility with Spouse Questionnaire: Development and Psychometric Property Evaluation

**DOI:** 10.30476/ijcbnm.2020.82160.1039.

**Published:** 2020-07

**Authors:** Maryam Nekoolaltak, Zohreh Keshavarz, Masoumeh Simbar, Ali Mohammad Nazari, Ahmad Reza Baghestani

**Affiliations:** 1 Department of Midwifery and Reproductive Health, School of Nursing and Midwifery, Tehran University of Medical Sciences, Tehran, Iran; 2 Midwifery and Reproductive Health Research Center, School of Nursing and Midwifery, Shahid Beheshti University of Medical Sciences, Tehran, Iran; 3 Department of Nursing and Midwifery, Shahroud University of Medical Sciences, Shahrood, Iran; 4 Physiotherapy Research Center,Department of Biostatistics, School of Paramedical Sciences, Shahid Beheshti University of Medical Sciences, Tehran, Iran

**Keywords:** Couple therapy, Factor analysis, Psychometrics, Sexual compatibility, Sexual satisfaction

## Abstract

**Background:**

Sexual compatibility between husband and wife is an effective factor in both sexual and marital satisfaction. However, there is limited valid and reliable questionnaire to measure the degree of sexual compatibility between the couples.

**Methods:**

In this exploratory mixed method study, 54 individuals were interviewed in the qualitative phase and 448 persons participated in the quantitative phase. Totally 502 participants (261 woman, 241 men) took part in this study. According to 205 final codes derived from the qualitative phase, 102 initial items were developed, the number of which reached 69 items after deletion and merging performed by the research team. After face validity, content validity and construct validity, 68 items were introduced into the construct validity phase.

**Results:**

Following exploratory factor analysis and promax rotation, the items were reduced to 35 in 4 factors: “Requirements of a sexual relationship”, “Sexual agreement”, “Contextual obstacles” and “Outcomes of sexual compatibility”. The questionnaire Cronbach alpha and correlation coefficient of the test-retest method were 0.90 and 0.91, respectively.

**Conclusion:**

Final Questionnaire included 35 items in 4 point-Likert scale with total score of between 35-140. This valid and reliable questionnaire is brief, easily interpreted and can measure the main factors affecting sexual compatibility with the spouse in clinics and research fields.

## INTRODUCTION

Sexual compatibility is one of the dimensions of sexual well-being in lifetime. ^1^ Sexual compatibility can lead to increasing sexual and marital satisfaction and joviality; ^2 , 3^ in contrast, sexual incompatibility may end up in divorce. ^4 - 7^ Sexual compatibility makes frequent replacement of sexual partner less likely and is effective in reducing sexual infections and in promoting sexual health of the individual and society. ^8 , 9^ Perceived sexual compatibility is a strong predictor of sexual and marital satisfaction. ^10^ Women with higher scores in sexual compatibility reported significantly less depression and higher levels of sexual motivation and desire. ^11^ Despite the importance of sexual compatibility in marital life, scant respective studies have been carried about sexual compatibility. ^10 - 13^ Moreover, limited valid questionnaires are available to measure sexual compatibility. 

Real knowledge of family relationships requires developing some methods for evaluation of couples and families. Science relies on the development of instruments. ^14^ Clinical practice on couples and families suffers from lack of measurement instruments and methods to follow the progress made in treatment. Common phrases such as "he/she will be back for treatment", "the couple seems satisfied" or "the couple is not divorced" cannot indicate the clinical performance of the therapist. In order to evaluate clinical performance, we need some questionnaires to document our measures. ^14^ Further, application of questionnaires to identify family problems is a time-saving practice. ^15^

Although Spanier questionnaire has been considered as a measurement instrument for dyadic adjustment in some literature, this questionnaire addresses all dimensions of compatibility in marital life, including income sharing, occupation, leisure time, education of children, etc., and only 2 out of 32 questions (6 and 29) are associated with sexual issues. ^16^

Hurlbert index of sexual compatibility (HISC) is a brief and simple questionnaire for measuring sexual compatibility, but some of its phrases are ambiguous. For example, the concept of sexual values, ideas and beliefs in items 1, 12 and 21 are interpreted differently in different people. In HISC, no qualitative study has been reported as a foundation for development of questionnaire, and advanced statistical methods of instrumentation (such as construct validity) are not used. The study population was limited to nurses and the diversity of sexual compatibility in the general population has not been studied. ^17^ In addition, sexuality is related to the culture of the community and changes over time, ^18^ so sexual compatibility factors in western society in 1993 may be different from those in eastern society three decades later. Therefore, developing a new questionnaire with modern statistical approach in Iranian society seemed necessary. The current study aimed to develop a valid and reliable questionnaire to inclusively evaluate sexual compatibility with the spouse. 

## MATERIALS AND METHODS

This methodological research is an exploratory sequential mixed method study that was carried out in two qualitative and quantitative phases during 2015 to 2017. The participants included 502 married men and women in formal marriage. Table 1 shows the number and sex distribution of participants in this study. The approval and code of ethics (SBMU2.REC.1394.73) was obtained from the research deputy of “Nursing and Midwifery School of Shahid Beheshti University”. In all phases of the research, written and oral informed consent was received from all participants. Prior to conducting qualitative interviews, voice recording permission was obtained. The researcher reminded the participants of her obligation to observe all ethical principles like secrecy, privacy, anonymity and permission to withdraw from the study. 

**Table 1:** Sex distribution of participants in this study

	Participants N (%)		Women N (%)	Men N (%)
Total	502 (100)		261 (52)	241 (48)
Qualitative phase	54 (10.75)		32 (6.3)	22 (4.3)
Quantitative phase	448 (89.24)	Item impact scores	7 (1.39)	5 (0.99)
		Content Validity	12 (2.39)	4 (0.79)
		Construct Validity	200(39.84)	200(39.84)
		Test- retest reliability	10 (1.9)	10 (1.9)

### *Qualitative Phase*

At first, the existing questionnaires were reviewed. None of them was qualified to be used in Iranian culture. Then, the qualitative study was conducted and the concept of sexual compatibility and factors affecting it were explored. The research setting for the qualitative phase included 2 clinics, 1 healthy house of municipality, and 1 religious forum (Heiat) in Tehran. Purposeful sampling was initiated and went on to reach data saturation. ^19^ Totally 54 participants (32 women and 22 men) took part in this phase. The inclusion criteria were women and men with at least one year of marital life, fluent in the Persian language, no major disease affecting their sexual performance (such as diabetes, spinal cord injury and substance abuse), willingness to participate in the study and ability to communicate and express their sexual life experiences. Data were collected through semi-structured in-depth interviews and written narratives which were analyzed using conventional content analysis with MAXQDA software version 10. After extracting the initial codes, again the review literature was used to complete the data. As the HISC was the most relevant and widely used questionnaire in the field of sexual compatibility, the final codes of qualitative study were compared with HISC. Almost all of the items in the Hurlbert questionnaire were somehow mentioned in our study and our qualitative study was comprehensive. Following frequent reviews and corrections, the preliminary questionnaire was developed and the second phase began.

### *Quantitative Phase*

In the second phase of this study, items of the questionnaire were designed based on the results of qualitative phase, and then face, content and construct validity evaluation process was conducted.

### *Face Validity*

In qualitative face validity, the level of difficulty, inappropriateness and ambiguity of the items was checked by all of the participants and some questions were changed. In quantitative face validity assessment, 12 participants (7 women and 5 men) were asked to score the importance of each item from 1 to 5. Next, the item impact score for each question was measured and decisions were made for deletion of those items with impact factor below 1.5. ^20^

### *Content Validity*

Qualitative and quantitative content validity was evaluated with the participation of 16 experts (12 women and 4 men) from different fields, including 8 reproductive and sexual health experts, 2 psychologists, 1 psychiatrist, 2 sociologists, 2 gynecologists and 1 nurse. Five experts had a history of instrument construction. In quantitative content validity, the specialists scored the necessity of each item within 1 to 3 ranges and accordingly, content validity ratio (CVR) was calculated and compared with Lawshe’s CVR table. Then, the specialists scored the relevance of each item from 1 to 4 and content validity index (CVI) was calculated according to the formula presented by Waltz and Bausel. ^21^

### *Construct Validity*

Construct validity was evaluated using exploratory factor analysis with the participation of 400 married men and women through SPSS 16 software. The research setting in construct validity included 21 healthcare, administrative, cultural and recreational centers and a family courthouse in Tehran. These people came from different ages, socioeconomic status, a variety of professions and educational levels with at least one month of formal marriage (Table 2). Convenience sampling was performed in the quantitative phase. Kaiser-Meyer-Olkin (KMO) index verified the adequacy of the samples. The number of factors was determined based on “scree plot”, and then decisions were made on omission of the items based on the degree of communalities. Finally, after Promax rotation, the scopes of the items were identified and named.

**Table 2:** characteristics of 400 participants in the construct validity phase

Variable		N (%)
Sex	Men	200 (50)
	Women	200 (50)
Marital life status	First marriage	355 (88.75)
	Divorced or on the verge of divorcing	28 (7)
	On the verge of marriage	13 (3.25)
	The second marriage	4 (1)
Occupation	Housewife	96 (24)
	Employee	186 (46.50)
	Self-employed	112 (28)
	Not mentioning the job	6 (1.5)
Duration of marriage	Shorter than 1 year	21 (5.25)
	1-10 years	155 (38.75)
	11-20 years	158 (39.5)
	21-30 years	52 (13)
	31-40 years	12 (3)
	Over 40 years	2 (0.5)
Education	High school educations	47 (11.75)
	Diploma	85 (21.25)
	Associate’s degree	46 (11.5)
	Bachelor	152 (38)
	Master	45 (11.25)
	Doctoral	8 (2)
	University student	17 (4.25)
Number of sexual relationships per month	.0	18 (4.5)
	1-3 times	70 (17.5)
	4-6	125 (31.25)
	7-9	67 (16.75)
	10-13	52 (13)
	14-16	16 (4)
	More than 16 times	16 (4)
	Not mentioning the number of relationships	36 (9)

### *Reliability*

Internal consistency and test-retest reliability of the questionnaire were confirmed. Twenty subjects (10 women and 10 men) participated in this part and responded twice to the test in a 14 day interval. 

## RESULTS

In this study, literature review was done in 4 stages: before, during and after the research, as well as before the results of the research were published. Studies were reviewed in Scopus, PubMed and Google Scholar databases. Here is the latest report on the Scopus database in 17 January 2020: 88 articles were obtained by searching sexual compatibility in article titles. After excluding the articles related to Agricultural, Genetic, Immunology and Computer Sciences, articles unrelated to heterosexual couples were omitted. Finally, 13 articles were retrieved. One of these 13 studies was qualitative ^22^ and the others were quantitative. In 7 out of 13 studies Hurlbert sexual compatibility index was used ^10 - 13 , 23 - 25^ and 3 studies were conducted usig researcher-made questionnaires or combination of several questionnaires. ^26 - 28^ Full text of 2 old articles was not accessed. ^29 , 30^ Foster had designed a 101-question questionnaire about sexual compatibility, ^29^ but it was not used in any of the subsequent studies and the text of the questionnaire was not accessible to any journal. Thus, the Hurlbert questionnaire is by far the most relevant and widely used questionnaire in the field of sexual compatibility and we will compare our questionnaire with Hurlbert’sThe current questionnaire is the product of a mixed methods study (qualitative and quantitative). In the qualitative phase, through interviewing 54 married women and men, the concept and factors affecting sexual compatibility were explored (Table 3). 

**Table 3:** Factors affecting sexual compatibility with spouse

Factors	Individual	Couple	Contextual
Facilitators	Awareness and preparation before marriage	Love each other	Passing of time
	Patience and tolerance	Practice, effort and experience	Asking for help from others
	Solving problems instead of quarrels	Sexual talk with spouse	Adorned wearing and making up at home
	Focus on own change	Female sexual orgasm	Leisure and travel
		Prominent role of husband	
		Sexual agreement	
		Mutual understanding	
		Developing a couple identity	
		Forgiveness and consideration of each other	
		Helping wife for babysitting	
		Humor in sex	
Inhibitors	Embarrassment	Lack of sexual talk with spouse	Lack of privacy for parents
	Bad body image	Ignoring spouse	Irregularities in the hours of sleep and wakefulness
	Self-centeredness	Challenge between parenting and partnering roles	Great attention to ceremony and luxury
	Taboo and the guilt of having sex	Challenge between spousal role and other roles	
	Dissatisfaction with the choice of spouse	Stingy and close-fisted husband	
		Fear of pregnancy	
		Problems of breastfeeding period	
Mediators	Marriage age	Couples sex solutions in specific fertility periods (menstruation, pregnancy, infertility treatment)	Woman tired of work outside home
	Religious rituals like bathing after sex (Ghusl)	The initiator of sex	Financial problems
	Sexual education		Social problems
	Sexual self-awareness		Watching sex movies

### *Concept and Factors Affecting Sexual Compatibility with the Spouse Based on Qualitative Study*

Sexual compatibility with the spouse is the couples’ participation for fulfilling each other’s sexual needs and solving problems arising from sexual discrepancies based on sexual understanding, sexual agreement and interest in continuing sexual relationships with the aim of mutual sexual satisfaction. ^22^

The factors affecting sexual compatibility included individual, marital and contextual factors that may play facilitating, inhibiting or intervening roles. ^31^

### *Item Generation*

Out of 905 preliminary codes in the qualitative study, 257 final codes were obtained according to which 102 items were developed. After omitting and merging by the research team, 69 items were left. 

### *Face Validity*

**Qualitative face validity:** The level of difficulty, inappropriateness and ambiguity of the items were examined by all of the participants and at the end, 10 questions were reworded. 

**Quantitative face validity (calculation of impact factor):** In this phase, the impact factor was calculated by 12 married men and women who were asked to score the importance of each item from 1 to 5 and the score of each item was calculated. One item (financial problems affect our sexual relationship) was scored below 1.5, but as qualitative participants emphasized it, this item was not omitted. Therefore, no item was omitted using the impact factor. 

### *Content Validity*

**Qualitative content validity:** In this section, 4 items were added, 2 were merged, and 1 was separated. Moreover, 4 items (sleeping together, pre-marriage friendship, spouse’s age difference, smoking alcohol and drug use) were transferred to the demographic section.

**Quantitative content validity:** In this part, CVR-CVI ratio was calculated. Three items gained low CVR ratio and were omitted. These items were as follows:

My education in childhood has affected my sexual relationship with my spouse (0.06). 

I’m worried that my husband becomes unfaithful to me (0.33). 

My husband’s friendship in cyberspace worries me (0.33).

Although the item *“We love each other”* (0.25) was not considered necessary by the experts and gained a low CVR score, the research team retained it in the questionnaire because the participants had stressed on it in the qualitative phase. Since there were 16 experts in our research and number 16 is absent in the Lawshe table, the number associated with 15 people, i.e. 0.49 was considered as the lowest score of items that was equivalent to agreement of 12 out of 16 experts (0.50). 

### *Construct Validity (Factor Analysis)*

After face validity and construct validity phases, 68 confirmed items were introduced to the construct validity phase and 400 participants (200 men and 200 women) answered the questions. 

KMO index for sampling adequacy was 0.908, indicating an excellent sampling adequacy. 

**Figure 1:**
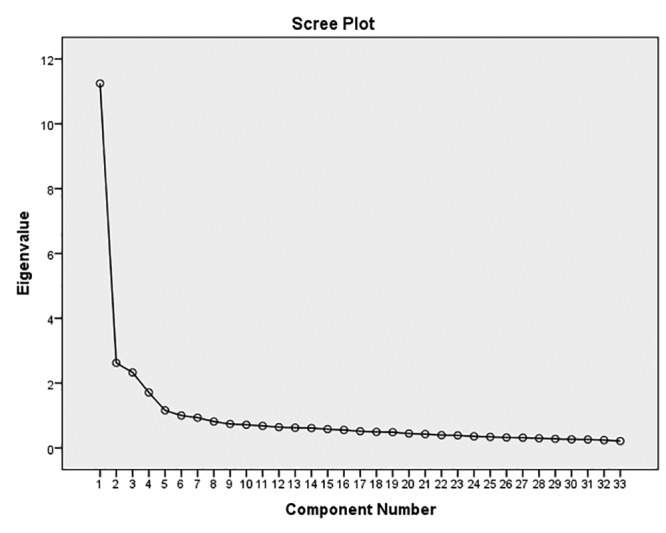
Scree plot

In the scree plot (Figure 1), four factors gained values higher than 1, so, SPSS settings were fixed according to 4 factors in the next phase. 

Based on the degree of communalities, all the items above 0.4 were retained. Two items close to 0.4 were also retained by the research team due to their qualitative importance. These two items included *“Talking without shame and embarrassment”* with communality of 0.38 and *“Improving the relationship with entertainment and traveling”* communality of 0.35. 

By considering the eigenvalues larger than 1 in the scree plot as the basis and communality above 0.4, finally four factors accounted for 53.109% of variance of total variability in sexual compatibility and the first to fourth factors explained 33.969, 7.505, 6.710 and 4.925 of variance, respectively (Table 4). 

**Table 4:** Total Variance Explained

Component	Initial Eigenvalues			Extraction Sums of Squared Loadings			Rotation Sums of Squared Loadings
	Total	% of Variance	Cumulative %	Total	% of Variance	Cumulative %	Total
1	11.889	33.969	33.969	11.889	33.969	33.969	11.534
2	2.627	7.505	41.475	2.627	7.505	41.475	5.691
3	2.349	6.710	48.185	2.349	6.710	48.185	3.978
4	1.724	4.925	53.109	1.724	4.925	53.109	3.077
5	1.222	3.492	56.602				
6	1.047	2.992	59.593				
7	0.954	2.726	62.320				
8	0.943	2.694	65.014				
9	0.770	2.199	67.213				
10	0.744	2.127	69.340				
11	0.711	2.031	71.371				
12	0.681	1.946	73.317				
13	0.652	1.864	75.181				
14	0.622	1.777	76.958				
15	0.607	1.734	78.693				
16	0.577	1.649	80.342				
17	0.567	1.621	81.963				
18	0.515	1.471	83.434				
19	0.495	1.415	84.849				
20	0.459	1.311	86.161				
21	0.448	1.280	87.441				
22	0.428	1.222	88.663				
23	0.395	1.128	89.791				
24	0.387	1.106	90.896				
25	0.378	1.081	91.977				
26	0.343	0.981	92.958				
27	0.325	0.927	93.885				
28	0.321	0.916	94.801				
29	0.303	0.865	95.666				
30	0.294	0.841	96.507				
31	0.277	0.792	97.299				
32	0.263	0.750	98.049				
33	0.254	0.727	98.776				
34	0.218	0.623	99.399				
35	0.210	0.601	100.000				

### *Definition of Sexual Compatibility with the Spouse Based on Construct Validity Results*

Sexual compatibility with spouse means the couple’s capability to provide the requirements of a sexual relationship, their agreement on how to have sex and their ability to manage the obstacles to achieve desirable outcomes. 

Following Promax rotation, those items with high correlation were included in a factor or subscale and each factor was given a name. The first factor was called “sexual compatibility requirements”, the second “sexual agreement”, the third “contextual obstacles”, and the fourth “outcomes of sexual compatibility”. 

### *Factors or Subscales of Sexual Compatibility with Spouse Questionnaire (SCSQ)*

**Factor 1: Requirements of sexual relationship:** Such requirements include the couple’s love for each other(Q1), improving sexual relationship over time(Q2), satisfaction with marriage (Q3), sexual attraction (Q4), sexual behavior (Q5), knowing each other sexually (Q6), reciprocal respect (Q7), mutual trust (Q8), active participation in sex (Q9), knowing each other’s body (Q10), wife’s understanding by the husband (Q11), sense of humor (Q12), flexibility (Q13), getting better sex in recreation and travel (Q14), dealing with the sexual needs of the spouse (Q15,16), solving sexual problems (Q17), and sexual talk (Q18). This subscale contains 18 items, one of which is reversed (Q16). The range of scores varies from 18 to 72 and a higher score indicates the couple’s capability to provide the requirements for sexual compatibility. Cronbach’s alpha for this subscales was 0.913. 

**Factor 2: Sexual agreement:** This agreement includes agreement on the frequency of sex (Q19), sex aids (Q20), having or not having anal and oral sex (Q21,22), watching or not watching sexual movies (Q23), contraceptive method (Q24), and sex position (Q25). This subscale included 7 items and the scores ranged from 7 o 28. A higher score conveyed a greater agreement. Cronbach’s alpha for this subscale was 0.833. 

**Factor 3: Contextual obstacles:** These obstacles include mismatch in sleeping and waking hours (Q26, 27), challenge between partnering and parenting roles (Q28, 29), and financial issues (Q30). This subscale comprises 5 items all of which are scored reversely. A higher score is indicative of the couple’s capability to handle the obstacles of their sexual relationship. Cronbach’s alpha for this subscale was 0.768

**Factor 4: Desirable outcomes:** These outcomes include become kinder (Q31), reach orgasm (Q32), mood change (Q33), importance of sex in life (Q34), and interest in the continuity of sex with spouse (Q35). This subscale included 5 items and the scores ranged from 5 to 20. A higher score conveyed better outcomes. Cronbach’s alpha for this subscale was 0.767

**Figure 2:**
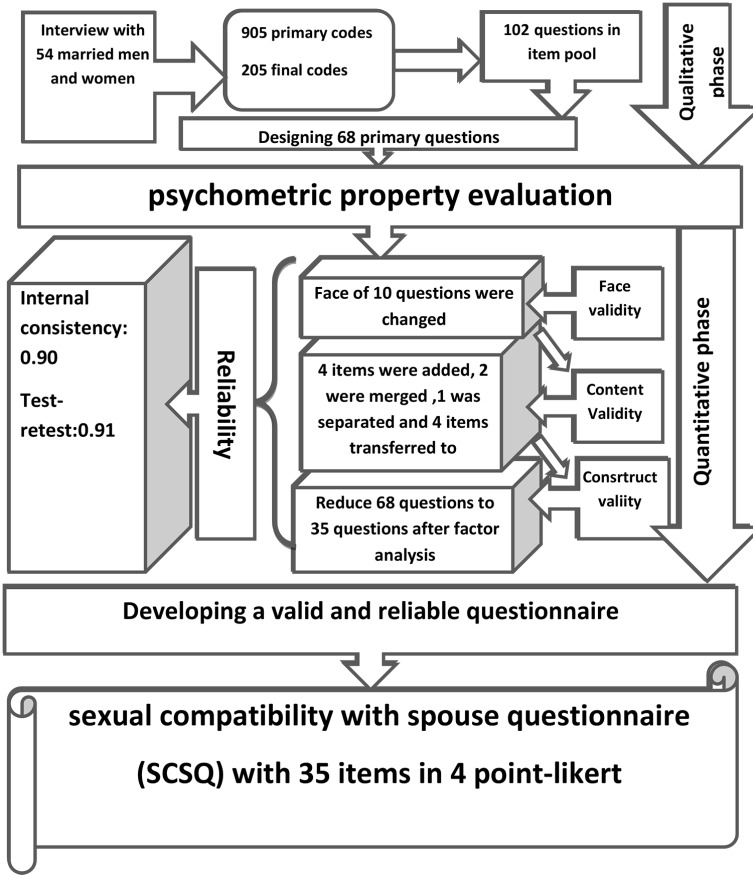
The stages of construction and psychometric property evaluation of sexual compatibility with spouse questionnaire (SCSQ)

Consequently, the final questionnaire included 35 items under 4 subscales with a 4 point-Likert scale, in which six items were reversed (Q16, 26, 27, 28, 29, 30). The scores range from 35 (the least compatibility) to 140 (the highest compatibility). The score of each subscale was calculated as the sum score of the items of that subscale. Then, they were converted to percentage and categorized into three levels of poor compatibility (0-33%), moderate compatibility (34-66%), and optimal compatibility (67-100%). Considering the questionnaires completed in the presence of the researcher, filling this questionnaire takes about 15 minutes. Table 5 presents the items and their quantitative values in different phases of psychometrics. Figure 2 shows stages of this study.

**Table 5:** Items of sexual compatibility with the spouse questionnaire and quantitative values of statements

Factor name /Cronbach’s Alpha	Item	Factor loading	Communality	CVI	CVR	Impact item
Factor 1: Requirements of sexual relationship 0.913	1-We love each other.	0.755	0.583	0.81	0.25	6.15
	2-Our sexual relationship improved over time.	0.768	0.590	0.88	0.94	1.99
	3-I’m satisfied with my marriage.	0.746	0.570	0.88	0.50	3.52
	4-My spouse is attractive for me sexually.	0.742	0.555	1	1	2.28
	5-I like my spouse’s sexual behaviors.	0.724	0.533	1	1	2.08
	6-I know my spouse’s sexual mood.	0.726	0.729	0.81	0.63	3.02
	7-We respect each other.	0.709	0.526	0.50	0.63	2.98
	8-We trust each other.	0.705	0.542	0.88	0.63	2.66
	9-We have mutual company and participation in our sex.	0.710	0.540	1	1	4.16
	10-We know each other’s body and sensitive sexual points.	0.688	0.478	1	1	4.29
	11-In our sex, husband cares about his wife’s spirit and needs.	0.667(1) 0.464(2)	0.487	1	1	4.23
	12-We have a sense of humor in our sex	0.657	0.445	0.88	0.50	1.80
	13-We are flexible in sex.	0.630(1) 0.515(2)	0.485	0.88	0.88	2.38
	14-Our sexual relationship gets better with recreation and travelling.	0.608	0.380	0.75	0.50	1.80
	15-My spouse understands my sexual needs.	0.611	0.391	1	1	2.84
	16-My spouse is indifferent to my sexual needs.	0.590	0.439	0.94	1	3.52
	17-We solve our sexual problems through due consideration and forgiveness.	0.563(1) 0.447(2)	0.381	1	1	2.84
	18-We talk about our sexual issues without shame and embarrassment.‌	0.552	0.363	1	1	2.66
Factor 2: Sexual agreement 0.833	19-We have agreement on the numbers of sexual relationship.	0.7059(1) 0.414(2)	0.533	1	1	2.26
	20-We have agreement on sex aids for intercourse (gel, spray and so on).	0.806	0.656	1	1	3.03
	21-We have agreement on having or not having anal sex.	0.797	0.659	1	0.88	2.24
	22-We have agreement on having or not having oral sex.	0.789	0.635	1	0.75	2.78
	23-We have agreement on watching or not watching sexual movies.	0.768	0.608	1	1	1.71
	24-We have agreement on contraceptive methods.	0.649	0.450	1	1	3.03
	25-We have agreement on the position of our bodies during sex.	0.559	0.436	1	1	2.57
Factor 3: Contextual obstales 0.768	26-Mismatch sleeping and waking hours among family members have faced our sexual relationship with problems.	2.93	0.88	0.94	0.88	2.93
	27-Mismatch sleeping and waking hours of me and my husband have faced our sexual relationship with problems.	2.84	1	1	0.636	0.769
	28-Taking care of children has made us neglectful of each other’s sexual needs.	0.695	0.518	0.94	0.50	2.81
	29-I’m worried my children imagine our sexual relationship.	0.689	0.570	0.81	0.50	1.80
	30-Financial problems affect our sexual relationship.	0.652	0.465	0.81	0.50	0.92
Factor 4: Desirable outcomes 0.767	31-After sex, we become kinder together.	0.670(1)	0.481	1	1	3.38
	32-I reach orgasm in sex with my spouse.	0.746(1) 0.421(4)	0.609	1	1	3.57
	33-Sexual relationship affects my mood and spirit.	0.849	0.532	0.88	0.75	1.55
	34-Sex is important in my life.	0.821	0.695	0.88	0.75	3.95
	35-I am interested in continuing my sexual relationship with my spouse for a lifetime.	0.599(1)	0.433(4)	0.88	0.63	4.09

### *Reliability*

Reliability was measured by internal consistency using Cronbach’s alpha and test-retest. Cronbach’s alpha for the entire questionnaire was obtained 0.90. Cronbach’s alpha of the subscales is presented in table 5. Correlation coefficient in the test-retest method was 0.91.

**Table 6:** Comparison of Hurlbert’s questionnaire with sexual compatibility with the spouse questionnaire

Title of questionnaire	Hurlbert’s sexual compatibility, 1993	Sexual compatibility with spouse, 2017
A qualitative study to design the questionnaire	Not reported	Reported (29)
Participants in construction phase of questionnaire	47 nurses (31 women, 16 men)	502 participants (261 women, 241 men)
Face validity and content validity	Not reported	Conducted qualitatively and quantitatively
Construct validity	Not reported	Conducted using exploratory factor analysis
Reliability	Cronbach’s alpha: 0.813	Cronbach’s alpha: 0.90
	Split half coefficient: 0.844	Test-retest coefficient : 0.91
Number of questions	25 items	35 items,
Scope of items	5 point-Likert	4 point-Likert
	14 reversed items	6 reversed items
Target group	There is no exact definition but it seems that it is specific to heterosexual couples.	Heterosexual couples with formal marriage.

## DISCUSSION

This mixed method study provided a comprehensive insight into the concept and factors affecting sexual compatibility. Based on the qualitative study, sexual compatibility with the spouse is couples’ participation for meeting each other’s sexual needs and solving problems arising from sexual discrepancies based on sexual understanding, sexual agreement and interest in continuing sexual relationships with the aim of mutual sexual satisfaction. According to the quantitative phase, sexual compatibility with the spouse means the couple’s capability to provide the requirements of a sexual relationship, their agreement on how to have sex and their ability to manage the obstacles to achieve desirable outcomes. Based on previous studies, sexual compatibility means having similar feeling to the partner in terms of sexual desires, behaviors, likes and dislikes ^32^ or similarity in emotional, cognitive, and behavioral components of a sexual relationship; ^33^ however, the current research suggests couple’s sexual companionship in dealing with sexual discrepancies as the foundation of sexual compatibility. Indeed similarity alone is not enough, the ability to manage differences and solving problems is also needed. In both qualitative and quantitative phases, agreement was the central basis of sexual compatibility. The aim of sexual compatibility is achieving mutual sexual satisfaction. Other researches also suggest that sexual compatibility is significantly related to sexual satisfaction ^10^ and sexual agreement. ^28 , 32^

As noted in the literature review, studies specifically focusing on sexual compatibility are limited. Most studies have been conducted on sexual compatibility in non-heterosexual couples or sexual adjustment after a psychological or physical trauma, whereas knowing sexual compatibility in a non-distressed couple is the basis for understanding sexual adjustment in unusual situations. Sexual adjustment may be used instead of sexual compatibility, but sexual adjustment usually refers to the sense of calmness or harmony by rearrangement of sexual relations after encountering a problem, for example sexual adjustment after spinal cord injury34 or sexual adjustment following treatment of cervical cancer. ^35^ Therefore, the terms “Sexual Adjustment” and “Sexual Compatibility” are not interchangeable. 

This study also aimed to develop a brief and reliable questionnaire. The only available questionnaire on sexual compatibility is the one developed by Hurlbert (1993). In comparison to HISC, SCSQ has a qualitative study for generating and designing the items; the number of participants in constructing the questionnaire is 10 times more than that of the Hurlbert’s questionnaire (502 participants compared to 47 participants), and the participants are from different classes and occupations while all those in HISC were nurses. Face, content and construct validities of SCSQ have been determined and reported. The latest statistical methods of construct validity, “exploratory factor analysis” have been used and it has categories and subscales. Also, it has a higher reliability. Table 6 shows comparison of Hurlbert’s questionnaire with sexual compatibility with spouse questionnaire.

The impact of contextual issues on the couple’s sexual compatibility was one of the unique points of this study. Factors such as mismatch in sleeping and waking hours (Q26, 27), challenge between partnering and parenting roles (Q28, 29), and financial problems (Q30) play as inhibitory factor on sexual compatibility. It could be due to cultural differences in the concept and experience of sexual compatibility in Iran and probably in similar eastern societies. Further studies with this questionnaire are required in different cultures.

This research resulted from the interaction of qualitative thinking and statistical analysis. In quantitative face validity, the item impact of “financial problems affect our sexual relationship” was below 1.5 and in quantitative content validity the item “we love each other” gained a low CVR score, but according to the research team opinion, these items were not omitted because they gained a high weight in the qualitative study. Finally, these items obtained a high factor loading in construct validity phase with 400 participants. Also, in the construct validity phase, two items “we talk about our sexual issues without shame and embarrassment” and “our sexual relationship gets better with recreation and travelling” had communalities below 0.4, but they were retained due to their importance in the qualitative phase. Other researchers also considered communality of 0.7 and higher good, between 0.4 and 0.7 fair and lower than 0.4 in need of review. ^36^

This questionnaire is associated with a critical issue which is so important for the respondents that they agreed to spend time to answer its items. Words and phrases in all items have been written in a simple and clear method because the items originated from in-depth qualitative interviews and the real life of people. Each item is related to a single concept. The items follow a psychological order and proceed from general to specific items. In construct validity, all 400 participants understood easily, answered the items with a great interest, and were satisfied with the order and fluency of the items. Also, its guideline is complete and clear and it can be easily interpreted. SCSQ seems to meet criteria of a suitable questionnaire. ^37^

There were no specific limitations to the research methodology. In the qualitative phase, data saturation was achieved in the sampling stage, and in the quantitative phase as far as possible individuals from different socioeconomic levels was sampled. However, given the cultural considerations of our society, obtaining the organizations’ approval to conduct interviews or fill out questionnaires on sexual issues was a time-consuming and challenging task, which in itself caused a diversity of sampling.

## CONCLUSION

The final questionnaire included 35 items under 4 subscales with a 4 point-Likert scale in which six items were reversed. This short and illustrative questionnaire is easily interpretable that measures the factors affecting sexual compatibility with the spouse and has been designed for heterosexual couples with formal marriage. This questionnaire is applicable for screening sexual compatibility in different populations and measuring sexual compatibility with the spouse before and after couple therapy sessions and sexual education interventions.
